# Choroid plexus transport: gene deletion studies

**DOI:** 10.1186/2045-8118-8-26

**Published:** 2011-11-04

**Authors:** Richard F Keep, David E Smith

**Affiliations:** 1 Department of Neurosurgery, R5018 BSRB, University of Michigan, Ann Arbor, Michigan 48109-2200, USA; 2 Department of Molecular & Integrative Physiology, R5018 BSRB, University of Michigan, Ann Arbor, Michigan 48109-2200, USA; 3 Department of Pharmaceutical Sciences, 4742C Medical Sciences II, University of Michigan, Ann Arbor, Michigan 48109-5633, USA

**Keywords:** transporters, channels, cerebrospinal fluid, knockout mice

## Abstract

This review examines the use of transporter knockout (KO) animals to evaluate transporter function at the choroid plexus (the blood-CSF barrier; BCSFB). Compared to the blood-brain barrier, there have been few such studies on choroid plexus (CP) function. These have primarily focused on Pept2 (an oligopeptide transporter), ATP-binding cassette (ABC) transporters, Oat3 (an organic anion transporter), Svct2 (an ascorbic acid transporter), transthyretin, ion transporters, and ion and water channels. This review focuses on the knowledge gained from such studies, both with respect to specific transporters and in general to the role of the CP and its impact on brain parenchyma. It also discusses the pros and cons of using KO animals in such studies and the technical approaches that can be used.

## Introduction

Choroid plexus (CP) epithelial cells and their linking tight junctions form the blood-CSF barrier (along with the arachnoid membrane) and are the primary site of CSF secretion. As with the cerebral endothelium, the site of the blood-brain barrier (BBB), the CP epithelium possesses a wide variety of transporters [1-4]. CP transporters are involved in cell and CSF homeostasis, the movement of nutrients into and waste products out of the CSF, and CSF secretion. In addition, as ependymal cells are not linked by tight junctions, CP transport may impact periventricular brain regions and potentially deeper structures by affecting CSF composition.

It is important to identify which transporters are present at the CP and their role (physiological and potentially therapeutic). However, the latter is complicated by the array of transporters present, overlapping substrate specificity and often a lack of specific inhibitors. One approach that has proven useful in transporter studies is the use of animals with gene deletions (e.g. KO mice) or gene mutations (functional knockouts). This approach has been used extensively in BBB studies to examine the role of different ATP-binding cassette (ABC) transporters in drug efflux (e.g. [5-8]). Multidrug resistance protein [MDR], breast cancer related protein [BCRP] and multidrug resistance associated proteins [MRPs] are all present at the BBB and have considerable substrate overlap. In comparison, relatively few CP transport studies have employed KO animals. This paper reviews those studies and the insights they give on CP transport and the relative functions of the blood-CSF and the blood brain barriers. It also discusses some methodological considerations when performing such studies. This paper does not aim to review the production of KO (and transgenic) mice.

### Studies of CP and blood-CSF barrier transport using KO animals

Multiple transporter KO mice have been generated. For example, there have been BBB transport studies on KO mice for Abcb1 (P-glycoprotein; Mdr1; [7]), Abcg2 (breast cancer related protein; Bcrp; [9]), Abcc-1 and -4 (multidrug resistance associated protein-1 and -4; Mrp-1 and -4; [10,11]), Oat3 and Oatp1a4 (two organic anion transporters; [11,12]) and Mct8 (a monocarboxylic acid transporter; [13]). Blood-brain barrier transport studies have also been performed on mice with naturally occurring mutations that result in transport inactivation (e.g. Octn2, an organic cation transporter, and Obr, a leptin transporter; [14,15]). Many more mouse KOs have been developed and used in transporter studies in non-brain tissues (e.g. kidney). As well as mice, there are also some naturally occurring functional knockouts in other species that have been used for BBB studies. Thus, there are Mrp2 KO rats (Eisai hyperbilirubinemic rat, [16]) and an Mdr1 KO in collie dogs [6] allowing species comparisons on the role of specific transporters. Currently, a number of transporter KO rats are under development, with the Mrp1 KO now being commercially available (SAGE Labs, St. Louis, MO, USA).

In contrast, the effect of transporter KO on CP has been the subject of far fewer studies and these are reviewed below. They provide insight into the types of CP experiments that can be performed, and their limitations, as well as information on the function of particular CP transporters. Broadly, the effects of gene deletion on transport has been mostly assessed *in vitro *using isolated CP, with limited studies on CP epithelial cell cultures because of the large number of mice required. *In vitro *studies have the advantage of enabling CP study without impact from other tissues (e.g. BBB, brain parenchyma). *In vivo*, most investigations have looked at CP transport and CSF entry after intravenous administration of compounds. Very few have used intracerebroventricular (icv) administration to study the effects of gene deletion on clearance from CSF.

**Figure 1:**
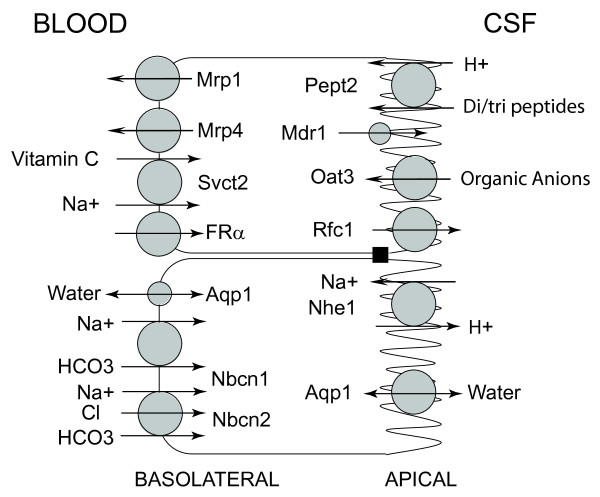
**Schematic of the CP with the distribution of the transporters discussed in this paper **[1,39,42]. For Aqp1, the distribution is primarily apical (CSF-facing) but some has been described on the basolateral membrane [1]. An apical and subapical distribution of Mdr1 has been reported [38]. However, it should be noted that the levels of Mdr1 at the CP are much lower than that found at the BBB.

The impact of the transporter KOs in relation to CP function is described in the sections below. Figure 1 gives the location of the different transporters at the CP.

#### Oligopeptide transport (Pept2)

Pept2 (SLC15a2) is a member of the proton-coupled oligopeptide transporter (POT) family which also includes Pept1, and two proton-coupled histidine transporters (Pht1, Pht2) [17,18]. All of these POTs transport small oligopeptides (2-3 amino acids) and peptidomimetics (e.g. alpha amino acid containing beta-lactam antibiotics), but only Pht1 and Pht2 can transport an amino acid, histidine [18]. Pept2 is present in a number of tissues including kidney, where it is involved in peptide reabsorption [19]. In brain it is present in the apical membrane of the CP epithelium but it is also present in neurons (neonate and adult) and astrocytes (neonate but not adult) [20]. It is not present at the BBB [20]. While both Pht1 and Pht2 are present in brain and CP, Pept1 is not [17,18,21,22]. Pht1 is present in neurons and small non-neuronal cells [22], but it is not known which brain parenchymal cells express Pht2.

**Figure 2:**
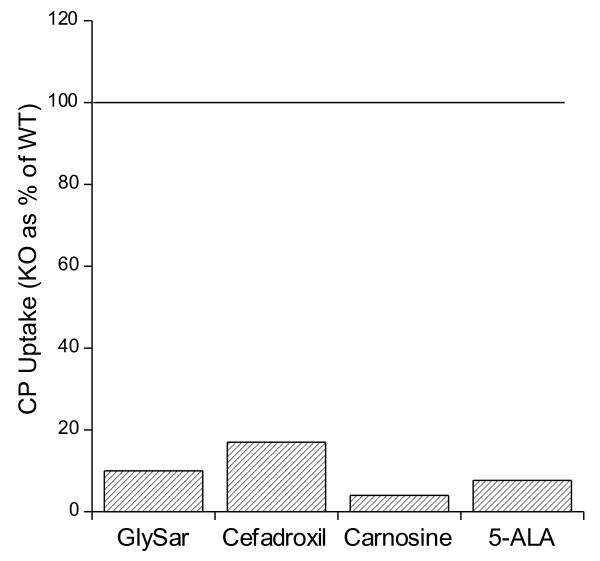
**The uptake of [^14^C]GlySar, [^3^H]cefadroxil, [^3^H]carnosine and [^14^C]5-ALA was measured in isolated CP from Pept2^-/- ^(KO) and Pept2^+/+ ^(WT) mice **[24-26]. The KO uptake is expressed as a % of WT. With the exception of cefadroxil (where a small portion of uptake is mediated by an organic anion transporter), uptake in CPs from KO mice was < 10% of WT.

To examine the functional importance of CP Pept2, we developed a Pept2 KO mouse (Pept2^-/-^) [23]. These mice are normal in appearance and body weight. We initially examined the uptake of glycylsarcosine (GlySar) into isolated choroid plexus [23]. GlySar is a hydrolysis resistant dipeptide that is a POT substrate. In the CPs from the Pept2^-/- ^mice [^14^C]GlySar uptake was reduced by ~90% compared to wild-type Pept2^+/+ ^animals [23,24] and we have since found similar major reductions for carnosine, another di-peptide, as well as for cefadroxil and 5-aminolevulinic acid (5-ALA), two peptidomimetics ([24-26]; Figure 2). In the case of cefadroxil there was evidence for a small component of uptake (10-15%) due to an organic anion transporter [25]. These results indicate that Pept2 is the predominant uptake transporter for di- and tri-peptides and small peptidomimetics in the isolated CP, a result further confirmed by the fact that L-histidine, a Pht1 and Pht2 substrate, did not alter uptake of these substrates.

**Figure 3:**
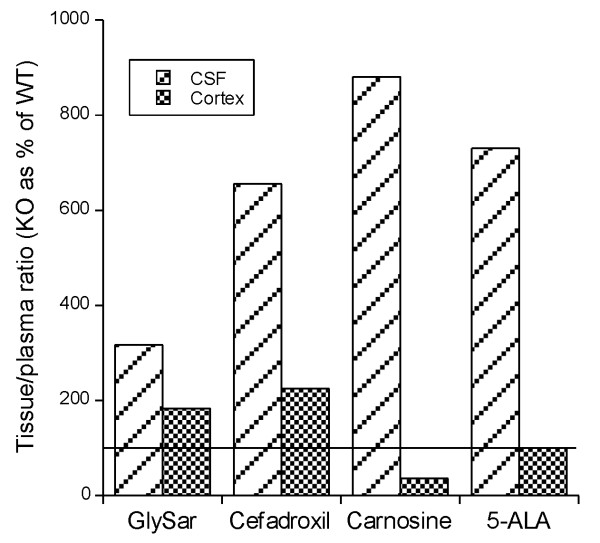
**The uptake of [^14^C]GlySar, [^3^H]cefadroxil, [^3^H]carnosine and [^14^C]5-ALA into CSF and cortex after intravenous administration into Pept2^-/- ^(KO) and Pept2^+/+ ^(WT) mice **[27-30]. For both genotypes, CSF/plasma and cortex/plasma ratios were calculated to account for differences in plasma concentrations which were lower in the KO mice because of reduced renal reabsorption of these peptides/peptidomimetics. The KO ratios were then expressed as a % of WT. Note the marked increase in CSF uptake for all 4 substrates in the KO while the effect on cortex was more modest (GlySar, cefadroxil), absent (5-ALA) or even reversed (carnosine).

The Pept2^-/- ^mouse has also allowed examination of the role of Pept2 in the distribution of potential substrates between blood, CP, CSF and brain *in vivo*. The first experiments examined GlySar, carnosine, cefadroxil and 5-ALA distribution after iv administration [27-30]. These experiments showed a marked increase in CSF uptake in the Pept2^-/- ^mice (Figure 3) as the transporter functions to clear substrates from the CSF into CP [18]. Interestingly, the effect of Pept2 on uptake into cerebral cortex was much smaller and more complex (Figure 3; see BBB vs. BCSFB discussion below). These experiments also served to emphasize one point about the use of transporter KO mice to study CP transport; that is, unless the transporter is expressed only in CP (or unless a tissue specific KO is used), there may be alterations in plasma substrate concentrations between the null and wild-type which necessitate referencing tissue concentrations to the plasma concentration. Even though kidney Pept2 caused wild-type mice to have higher plasma concentrations of Pept2 substrates after iv injection (e.g. [29]), these concentration changes did not account for the differences in CSF and CP concentrations between the two genotypes.

**Figure 4:**
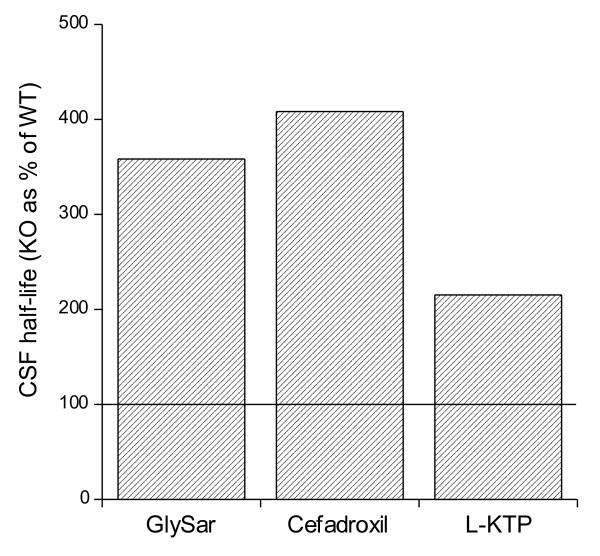
**Comparison of the carrier-mediated clearance of [^14^C]GlySar, [^3^H]cefadroxil, L-[^3^H]kyotorphin (L-KTP) from CSF after intracerebroventricular administration to Pept2^-/- ^(KO) and Pept2^+/+ ^(WT) mice **[31,32]. A half-life for the clearance of these compounds (corrected for diffusion using radiolabeled mannitol) was calculated and the KO values expressed as a % of WT. Note the prolonged half-life for each of the compounds in the KO mice reflecting reduced CP-mediated clearance.

The Pept2^-/- ^mouse has also been used to look at substrate distribution after intracerebroventricular (icv) administration. These studies have shown that the clearance of GlySar, cefadroxil and L-kyotorphin (an endogenous analgesic dipeptide) from CSF is reduced in Pept2^-/- ^mice compared to wild-type mice and that this effect is matched by reduced CP uptake in the null mice [31,32] (Figure 4). Using autoradiography, we studied the impact of Pept2 deletion on penetration of [^14^C]GlySar into periventricular brain in adult mice. These studies are complicated by the presence of Pept2 in some neurons but did show that there is a rapid drop-off in GlySar (MW = 146) concentration with distance from the CSF system [32]

The Pept2^-/- ^mice show no obvious phenotypic differences from the Pept2^+/+ ^mice under normal conditions (e.g. in growth or behavior). However, we have found that the null mice are more susceptible to 5-ALA-induced neurotoxicity after systemic administration (as might be expected from the higher 5-ALA CSF concentrations [27]). Intracerebroventricular administration of L-kyotorphin also induces more analgesia in the null mice, as might be expected from the reduced clearance of this drug from CSF into CP [31].

#### ATP-binding cassette (ABC) transporters

The ABC transporters have been studied extensively at the BBB using KO mice and these transporters markedly reduce the penetration of a wide variety of drugs from blood to brain. Thus, as shown by Schinkel and colleagues, deletion of P-glycoprotein markedly increases the entry of a wide variety of drugs into brain [7,33]. Similarly, Bcrp KO can significantly impact the brain uptake of specific drugs [9]. For Mrp-1 and -4 KOs there are some more modest effects [10,11]. It should be noted that under normal housing and dietary conditions, Mdr1, Bcrp, Mrp1 and Mrp4 KO mice are normal in appearance, body weight and fertility [7,34-36].

Data from KO animals, supported by immunohistochemistry, have shown that CP epithelial cells differ from cerebral capillary endothelial cells in the distribution of ABC transporters and, therefore, the effects of transporter KO. Thus, Shen *et al*. [37] found that while Mdr1a/b KO caused increased penetration of topcotecan into brain parenchyma, it causes reduced penetration into ventricular CSF [37]. They also found that Bcrp KO mice had increased penetration of the drug into brain parenchyma but reduced penetration into CSF. The effects of Mdr1a/b and Bcrp deletion were additive at both sites. These differential effects of the KOs on drug entry at the blood-CSF and blood-brain barriers are due to the two ABC transporters being on the CSF-facing membrane at the CP and at the blood-facing membrane at the cerebral endothelium [37-39].

In contrast to the Shen *et al*. [37] study, Doran *et al*. [40] found marked increases in the brain and CSF entry of metoclopramide, risperidone and 9-OH-risperidone in Mdr1a/b KO mice compared to WT, although the effects on CSF were less than on brain. A potential reason for the difference may be the site of CSF sampling. Whereas Shen *et al*. [37] sampled ventricular CSF adjacent to the CP by microdialysis, Doran *et al*. [40] sampled bulk CSF from the cisterna magna where there may have been more BBB influence as the CSF flows past brain tissue. Interestingly, Mealey *et al*. [6] examined brain and CSF penetration of ^99 m^Tc-sestamibi, a Mdr1 substrate, in WT dogs and dogs with a mutation in Mdr1 and found that while brain levels were elevated with the mutation, CSF levels were unchanged. In this study, CSF was sampled from the cisterna magna which may have influenced the results.

It should be noted that the relative impact of Mdr1 at the BBB and BCSFB depends upon not only cellular localization but also the amount of the transporter. Thus, the amount of Mdr1 at rat and human CP is much less than that found in cerebral microvessels, as assessed by Western blot [41]. This is in marked contrast to another ABC transporter, Mrp4, where the expression is much higher in CP than at the BBB [41].

In contrast to Mdr1 and Bcrp, Mrp4 is thought to be on the basolateral membrane of the CP epithelium and the luminal membrane of the cerebral endothelium, i.e. the transporter at the two sites may work in concert to keep CNS levels low [39]. Thus, Leggas *et al*. [35] found that Mrp4-deficient mice had much greater entry of an anti-cancer agent, topotecan, into CSF and brain than WT mice.

Mrp1 is on the basolateral membrane of the CP epithelium involved in clearing substrates from the CP to blood [39,42]. Thus, Wijnholds *et al*. [43] found that CSF levels of etoposide were increased 10-fold by Mrp1 KO. However, Lee *et al*. [16] found no difference in the efflux of estradiol-17β-glucuronide and 2,4,-dinitrophenyl-S-glutathione from CSF. This may reflect the relative importance of Mrp1 for these compounds compared to other efflux mechanisms. Lee *et al*. [16] also examined the transport of estradiol-17β-glucuronide and 2,4,-dinitrophenyl-S-glutathione in the Mrp2 deficient Eisai rat and found no effect on clearance from the CSF. This may be due to the very low or negligible expression of Mrp2 at the CP epithelium [16].

#### Organic anion transport

There are multiple organic anion transporters at the blood-CSF and blood-brain barriers, including (but not limited to) members of the SLC22 (Oat) and SLC21/SLCO (Oatp) families [42,44]. These transporters have overlapping substrate affinities making it difficult to assign the relative role of individual transporters. Sweet *et al*. [45,46] developed an Oat3 KO mouse to examine the role of this transporter. The null mice are normal in appearance and are fertile [45]. Oat3 is present on the apical membrane of the CP epithelium (Figure 1) where it is involved in clearing organic anions from CSF. In isolated CP, para-aminohippuric acid and fluorescein uptake was essentially abolished in Oat3 null mice, whereas the uptake of estrone sulfate was only reduced by a third and taurocholate uptake was unaffected [45,46]. Thus, the importance of Oat3-mediated transport at the CP depends on the nature of the organic anion. In addition, it is still uncertain as to the importance of blood-CSF vs. blood-brain barrier organic anion transport as the Oat3 KO mouse has not been used for *in vivo *CP studies.

#### Transthyretin (TTR)

TTR is the protein most abundantly synthesized and secreted by the CP. TTR binds thyroxine (T_4_) and retinol and it has been hypothesized that TTR is involved in the transport of T_4 _from blood to brain. Palha *et al*. [47,48] examined this hypothesis in TTR null mice. They found no difference in the rate of [^125^I]T4 entry into the brain in TTR-null mice. They also found that cortex, cerebellum and hippocampus T4 levels were similar in the TTR-null and WT mice. However, CP T4 levels were very markedly lower in null mice (14% of WT). Thus, TTR effects on T4 are probably limited to the CP-CSF system and areas adjacent to that system. Interestingly, Richardson *et al*. [49] found that apoptosis in neural stem cells/progenitor cells in the subventricular zone was reduced in TTR null mice to a similar extent to that found in hypothyroid mice.

#### Sodium-dependent vitamin C transporter-2

Ascorbic acid is not synthesized in the brain and is, therefore, reliant on transport from blood [50]. The sodium-dependent vitamin C transporter-2 (svct2; slc23a1) is highly expressed at the CP [51] where it is present at the basolateral membrane transporting ascorbic acid from blood to CP (Figure 1) [52]. Svct2 appears to be essential for this transport as Svct2 KO mice have undetectable ascorbic acid levels in the brain at birth and die perinatally [53]. As Svct2 is highly expressed at the CP but absent from the BBB [50] this suggests that the very low brain ascorbic acid levels in the KO are due to the lack of the CP transporter.

#### Folate transport

Some of the complexity of using transporter KO mice to study CP function is demonstrated by folate transport. Folate transport from blood to CSF at the CP is thought to involve Folate Receptor alpha (FRα) at the basolateral membrane and the Reduced Folate Carrier 1 (Rfc1) at the apical membrane [50]. KO mice have been produced for both FRα and Rfc1 but both are embryonic lethal with the embryos showing severe abnormalities [54,55]. Even though there is some embryo rescue by giving pregnant dams folic acid, RFC null neonates are markedly growth retarded and have multiple compromised organ systems [55]. The absence of adult KO animals make transport studies difficult while the effects on multiple organ systems make it difficult to assess whether any effects on brain are direct or indirect. Analysis of folate transport at the CP would be greatly facilitated by the generation of CP-specific KO mice.

#### Ion transporters/channels

The CP has a wide array of ion transporters and channels [1] that are involved in CSF and cellular ion homeostasis, some of which have been examined using KO mice (the Na^+^/H^+ ^exchanger, Nhe1; the Na/bicarbonate cotransporters, Nbcn1 and Nbcn2; Figure 1). Changes in the CSF composition of a number of ions would affect neuronal excitability. Thus, the CSF composition of potassium and calcium are regulated very tightly during changes in plasma composition [56,57]. Similarly, CSF pH is also substantially regulated during changes in blood pH (with the exception of acute respiratory changes). The CPs are also the primary site of CSF secretion which involves CP ion transporters and channels [1]. Understanding which transporters are involved in CSF secretion may generate targets to treat raised intracranial pressure and hydrocephalus and KOs have been used to try and define the roles of specific transporters. It should be noted that the transporters present at the CP are not tissue specific. Many ion transporter KOs result in significant developmental effects on the animals. For example, in relation to the KOs described below, Nhe1 KO mice have ataxia, seizures and growth retardation [58,59], Nbcn1 KO mice develop blindness and auditory problems [60] and Nbcn2 KO mice mostly die around weaning unless special care is taken with feeding [61]. With such profound effects, it is often possible that there are secondary and compensatory effects of the transporter deletion.

Two Na/bicarbonate cotransporters (Nbc), Nbcn1 and Nbcn2 are present at the CP basolateral membrane [62]. Targeted deletion of Nbcn2 (Slc4a10) resulted in reduced ventricular size, that probably results from reduced CSF production [61]. In addition, the CP epithelial cells from these KO mice also have altered pH handling, although there are some compensatory changes in the Na^+^/H^+ ^exchanger, Nhe1 [63].

Nhe1 is normally located on the CP apical membrane where it is involved in H^+ ^excretion [1]. In Nhe1 KO mice, acid extrusion (in the absence of CO_2_/HCO_3_^-^) from the CP was almost completely abolished, demonstrating the importance of this transporter (rather than other Nhe isoforms) in epithelial cell pH regulation [63]. Interestingly, Nhe1 KO did not change the expression of Nbcn1 or Nbcn2 in the CP [63].

Speake *et al*. [64] have used patch clamp to examine CP from mice deficient in a chloride channel, Clc2. They found that the loss of this channel did not alter the inward-rectifying anion conductance in CP epithelial cells, a conductance thought to be important in CSF secretion.

#### Aquaporins

A water channel, aquaporin 1 (Aqp1), is highly expressed at the apical membrane of the CP epithelium (Figure 1). Aqp1 null mice appear normal apart from a mild growth retardation but they are unable to create a hypertonic urine during water deprivation [65]. Oshio *et al*. [66] compared the water permeability of choroid plexus from Aqp1 null and wild-type mice and found a five-fold reduction in the former. Aqp1 null mice also had reduced CSF production (~25%) and intracranial pressure. There are currently no specific inhibitors for aquaporins and, therefore, knockout mice are an important method for elucidating their function.

### Methodological considerations

As noted above, almost all (if not all) transporters are expressed in more than one cell type. Thus, care must be taken in designing experiments and interpreting results from KO animals (e.g. a transporter KO may affect blood levels of a substrate as well as CP transport). This is particularly the case when assessing a non-transporter phenotype (such as behavior or neurotoxicity). A method of limiting such concerns is to use cell-specific KO mice [67,68], but these have not, as yet, been used for CP transporter studies.

Another concern is potential compensation for a transporter KO. As there can be considerable substrate overlap between different transporters, it is possible that the loss of one transporter may be compensated for by the upregulation of another. We have examined this possibility in the Pept2^-/- ^mouse and found no mRNA or functional upregulation of the other POT family members at the CP [29] but this may not be the case for other transporters. Thus, deletion of the Na/bicarbonate transporter, Nbcn2, alters the CP location of the Na^+^/H^+ ^exchanger, Nhe1, a transporter that is also involved in pH regulation [63]. One potential method for alleviating this problem is to use conditional KOs where the gene of interest can be knocked out at a specific time in the animal's life, lessening the chance of adaptive changes that might occur during development [67,68]. Such temporal conditional KO mice have, as yet, not been used in CP transporter studies.

Another approach to lessen potential compensatory effects of knocking out a gene is to acutely knock down gene expression with a small interfering RNA (siRNA). This approach is easier *in vitro *than *in vivo *(siRNA delivery). It has been used by both Wang *et al*. [69] to knock down the expression of the divalent metal transport 1 (Dmt1) in a choroid plexus epithelial cell line and by Boassa *et al*. [70] to knock down aquaporin 1 in primary choroid plexus epithelial cell cultures.

### BBB *vs*. BCSFB

The importance of CP transport to brain parenchyma has long been debated. For D-glucose, the high rate of transport by BBB Glut-1 and the proximity of the vasculature to neurons results in CP D-glucose transport having a very minor role in the uptake of this essential nutrient into brain. In contrast, for ascorbic acid, CP transport (via the sodium dependent vitamin C transporter, Svct2) plays a predominant role with brains of Svct2 KO mice having almost no ascorbic acid [50]. The role the CP plays in the transport of nutrients, waste products or pharmaceuticals will depend on the transporters present at the BCSFB, BBB and parenchymal cells, the passive diffusion of those compounds across the barriers and the relative distance of the parenchymal cells to the ventricular system. KO animals give a methodology to examine the role of CP transport, although care is needed in interpreting the data based on the location of the deleted transporter (CP alone, CP+BBB, CP +parenchymal cells), the size of the brain (which will effect penetration from the CSF into parenchyma) and the target site (periventricular *vs*. distant from ventricular system) as outlined below.

We have examined the effect of Pept2 KO on cerebral cortex penetration of GlySar, cefadroxil, carnosine and 5-ALA after intravenous administration. While the accumulation of these drugs in CSF is markedly increased (300-900%; Figure 3), the effects on cerebral cortex were more modest or even the reverse to that found in CSF (Figure 3). We examined these findings further using icv injections of [^14^C]GlySar and autoradiography [32]. Even though GlySar has a molecular weight of 146 and ependymal cells are not linked by tight junctions, there was a rapid decline in tissue [^14^C]GlySar levels with distance from the ependyma in both Pept2-/- and WT mice with concentrations dropping by 50% over 0.53 and 0.34 mm, respectively. Thus, the impact of changes in CSF concentrations resulting from a loss of a CP transporter was likely to be limited to the periventricular zone. Milhorat *et al*. [71] examined the penetration of radioactive sodium from the CSF system into brain in rhesus monkey after an intravenous infusion and they also found a steep decline in concentration as tissue was sampled further away from the ependymal and subarachnoid space.

It should be noted that in our studies on the effects of Pept2 KO on the distribution of peptides into CSF and brain parenchyma, described above, we examined the impact after a single injection (iv or icv) to mimic drug administration. The effects of CP transporter KO on brain parencyhma may be greater for compounds normally found in the body. Thus, as noted above, Svct2 KO mice have very low brain vitamin C levels [53] that is attributable to the loss of blood to CSF transport at the CP. With a naturally-occurring compound there may be chronic changes in CSF levels in the transporter KO with a greater impact on brain parenchyma.

Some of the potential difficulties in using whole animal KO animals to assess the effects of CP transport on brain parenchyma are exemplified by the effects of such KOs for the ABC transporters. Mrp4 is present at the CP and the BBB and in both tissues it is involved in transporting substrates back to blood [39,42] making it difficult to attribute effects on CSF and brain parenchyma to one barrier site alone. Mdr1 and Bcrp are also present at both the CP and BBB, but they are on the CSF (brain)-facing membrane at the CP and the blood-facing membrane at the BBB [39]. This should give an opportunity to examine the impact of the two sites of transport which have opposing effects. However, care is necessary as the balance will be intimately related to sampling sites, i.e. parenchymal distance from the CSF system and the distance of the CSF sampling site from the CPs.

In general, the extent of the impact of a CP transporter knockout on the parenchyma (and periventricular zone in particular) will depend upon multiple variables. It will depend upon the magnitude of the KO effect on substrate CSF levels, the clearance via CSF flow, the rate of penetration into parenchyma across the ependymal, trapping or exclusion of the substrate from parenchymal cells, and the rate of diffusion and/or transport across the BBB. Compensatory transporters (in the absence or presence of upregulation) will also serve to confound this issue.

## Conclusions

Transporter KO animals are a useful tool in elucidating the functional role of CP transporters. This is particularly the case as we gain more understanding of the overlapping substrate specificities of different transporters and the possible effects of inhibitors on multiple transport systems. As yet, only the effects of whole animal transporter knockouts on CP transport have been examined. Under normal conditions the transporter KOs examined had no obvious phenotype associated with the CP transporter deletion, with the exception of Svct2. However, such deletions can have marked effects on the CSF levels of substrates, drug efficacy and substrate-induced neurotoxicity. The effects of CP transporter KOs on brain parenchyma substrate delivery are variable with the greatest effects on the periventricular zone rather than the deep brain structures.

## List of abbreviations

ABC: ATP-binding cassette; 5-ALA: 5-aminolevulinic acid; BBB: blood-brain barrier; BCSFB: blood-CSF barrier; Bcrp: breast cancer related protein; CP: choroid plexus; CSF: cerebrospinal fluid; FRα: folate receptor alpha; Rfc1: reduced folate carrier 1; GlySar: glycylsarcosine; KO: knockout; Mdr: multidrug resistance protein; Mrp: multidrug resistance associated protein; POT: proton-coupled oligopeptide transporter; siRNA: small interfering RNA; T_4_: thyroxine; WT: wild-type.

## Competing interests

The authors declare that they have no competing interests.

## Authors' contributions

RFK and DES co-wrote this manuscript. All authors read and approved the final manuscript.

## Authors' information

RFK is Professor of Neurosurgery and Molecular & Integrative Physiology at the University of Michigan. DES is Professor of Pharmaceutical Sciences.
